# Highly-accurate metabolomic detection of early-stage ovarian cancer

**DOI:** 10.1038/srep16351

**Published:** 2015-11-17

**Authors:** David A. Gaul, Roman Mezencev, Tran Q. Long, Christina M. Jones, Benedict B. Benigno, Alexander Gray, Facundo M. Fernández, John F. McDonald

**Affiliations:** 1 School of Chemistry and Biochemistry, Georgia Institute of Technology, Atlanta GA 30332 (USA); 2 School of Biology, Integrated Cancer Research Center, Georgia Institute of Technology, Atlanta GA 30332 (USA); 3 College of Computing, Georgia Institute of Technology, Atlanta GA 30332 (USA); 4 Ovarian Cancer Institute, Atlanta GA 30342 (USA); 5 Parker H. Petit Institute of Bioengineering and Biosciences, Georgia Institute of Technology, Atlanta GA 30332 (USA)

## Abstract

High performance mass spectrometry was employed to interrogate the serum metabolome of early-stage ovarian cancer (OC) patients and age-matched control women. The resulting spectral features were used to establish a linear support vector machine (SVM) model of sixteen diagnostic metabolites that are able to identify early-stage OC with 100% accuracy in our patient cohort. The results provide evidence for the importance of lipid and fatty acid metabolism in OC and serve as the foundation of a clinically significant diagnostic test.

Ovarian cancer (OC) is the most lethal of all gynecological malignancies and the fifth leading cause of death among women living in the United States^1^. The disease is essentially asymptomatic until late stages when the 5-year relative survival rate is <44%^2^. If detected and treated early in its progression, the 5-year survival rate is ~90%. For this reason, considerable effort has been focused on the development of a screening test to diagnose OC early in its progression^3^. This challenge is confounded by the fact that because the disease is in low prevalence in the general population (~0.1% in USA), a screening test must attain a positive predictive value (PPV) of >10%, with a specificity ≥99.6% and a sensitivity ≥75% to be of clinical relevance in the general population^4^.

The current standard screening method for OC involves trans-vaginal ultrasound and measurement of serum CA-125 levels^3^. Combined, these tests result in a positive predictive value of only 24%^5^. A recent study reports that monitoring changes in serum levels of CA-125 over time rather than reliance on a single predefined threshold level of significance can increase accuracy of detection up to 86%^6^. In addition, a variety of proteomic^7^ and microarray^8^ based tests are currently under development but, thus far, no assay has attained the stringent level of accuracy required to be of clinical relevance in the general population.

We report here on the combined use of ultra-performance liquid chromatography, high-resolution mass spectrometry (UPLC-MS) and tandem MS (MS/MS), combined with a customized support vector machine (SVM)-based learning algorithm for identification of 16 diagnostic metabolites that collectively are able to distinguish early-stage OC with 100% accuracy in our cohort. The results provide the foundation for clinically-significant diagnostic tests and evidence for the importance of alterations in lipid and fatty acid metabolism in the onset and progression of the disease.

## Results

Negative ion mode UPLC-MS interrogation of the serum metabolome from 46 early stage (I/II) serous epithelial ovarian cancer (EOC) patients and 49 age-matched normal healthy controls (Supplementary Table S1) resulted in the detection of >4000 spectral features (R_t_, m/*z* pairs). After filtering and curation to remove inconsistent and/or ambiguous features, a remaining pool of 255 (Supplementary Table S2) was used to build a discriminant linear support vector machine (SVM) model that was evaluated by leave-one-out cross-validation (LOOCV)^9^. Binary classifications (cancer/normal) were established through a previously described “metabolic score” decision function that numerically separates the predicted cancer (positive score) from control (negative score) samples^10^.

**Figure 1:**
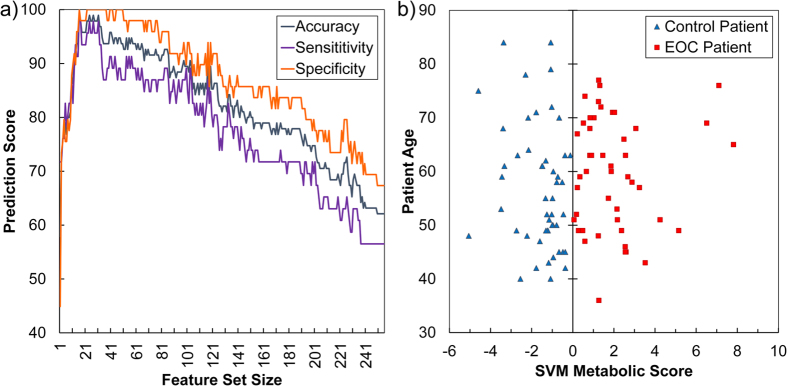
Recursive feature elimination (RFE) selects 16 metabolic features that distinguish early stage serous epithelial ovarian cancer (EOC) serum samples with high accuracy. (**a**) Evolution of accuracy using support vector machine (SVM)-RFE feature selection for metabolic classifiers. The initial 255 metabolic features identified by ultra-performance liquid chromatography-mass spectrometry (UPLC-MS) provided only moderate predictive accuracy (accuracy 62%; specificity 57%; sensitivity 67%) in distinguishing EOC from control samples. SVM-RFE selected a minimum of 16 metabolic features that provided 100% accuracy (100% sensitivity; 100% specificity) in distinguishing between EOC and control samples. **(b)** Visualization of the optimal separation between EOC and control samples by the SVM model. The X-axis is the optimal weight vector of the SVM model; the Y-axis is the age of donors (EOC patients or normal control women at the time of sample collection). The vertical line is the projection of the separating hyperplane generated by the SVM model. The discriminant linear SVM model was evaluated by leave-one-out cross-validation (LOOCV).

Using all 255 metabolic features, a first SVM model was generated displaying moderate predictive accuracy (accuracy 62%; specificity 57%; sensitivity 67%). Since SVM models built upon large datasets typically contain uninformative features, a number of feature selection methods have been developed to identify subsets with optimal predictive accuracy^11^. We employed a previously described recursive feature elimination (RFE) method^9^ to select features that distinguished the early-staged EOC samples from controls with optimal accuracy. As shown in the Fig. 1 (see also Supplementary Figs S1–2), 100% accuracy (100% sensitivity, and 100% specificity) was obtained with a minimum of 16 features. The high predictive accuracy of these 16 metabolites was independently validated by orthogonal partial least squares-discriminant analysis (oPLS-DA) using a variety of cross-validation approaches (Supplementary Table S3, Supplementary Figs S3–4).

**Figure 2:**
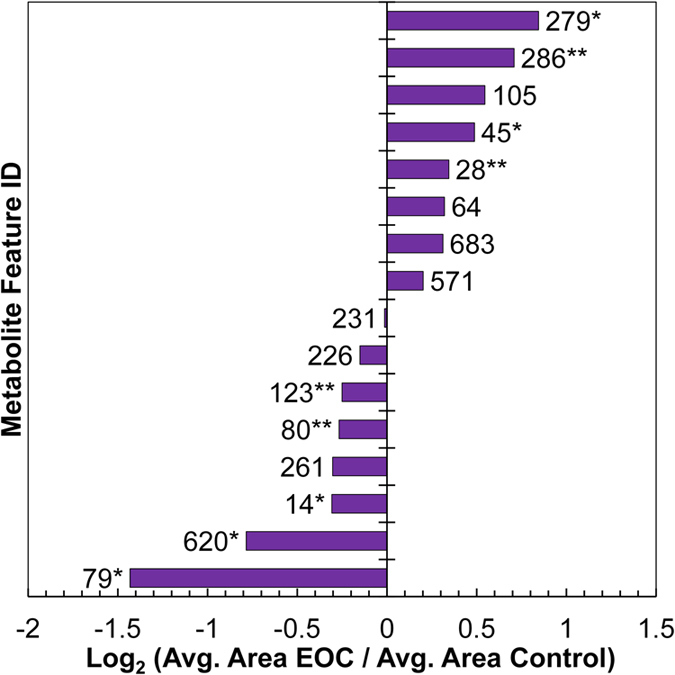
Fold-change of average peak areas of each discriminant feature. Positive values indicate higher levels of metabolite observed on average for EOC patients compared to control patients, while negative values indicate inverse relationship (*p < 0.05; **p < 0.10, Mann Whitney U test).

The high resolution MS technology employed allowed generation of accurate masses and isotopic patterns for each discriminant feature and therefore establishment of candidate elemental formulas. These proposed metabolite identities were confirmed by UPLC-MS/MS, and the resultant tandem MS spectra were compared to those in databases or literature, resulting in chemical identification of 11 of the 16 discriminating features (Table 1, Supplementary Fig. S5, Supplementary Table S4). Two feature identities were further supported by comparison to a standard (R_t_ and ion fragmentation pattern). Relative concentration levels of about half of the 16 features were elevated and half reduced in cancer samples relative to controls (Fig. 2).

Many of the identified features were lipids or fatty acids. An emerging body of evidence has implicated changes in lipid and fatty acid metabolism with the onset and progression of ovarian^12^ and other types of cancer^13^. In many cases, these changes have been linked to the aberrant expression of genes involved in lipid/fatty acid synthesis. For example, the well-known tumor suppressor gene p53 is mutated in >95% of high-grade serous ovarian cancers^14^. It has recently been reported that the protein encoded by p53 (TP53) interacts with sterol regulatory element-binding proteins (SREBPs) and guanidinoacetate N-methyltransferase (GAMT) resulting in the elevated expression of enzymes involved in fatty acid and cholesterol biosynthesis and the inhibition of fatty acid oxidation leading to lipid anabolism and accelerated tumor growth and progression^15^.

Two of the identified metabolites are lysophospholipids (LPLs) [lysophosphatidylethanolamine (LPE) and lysophosphatidylinositol (LPI)]. Serum levels of LPLs have been previously reported to be elevated in OC patients and in matched sets of samples isolated from preoperative vs. postoperative patients^16^. LPIs are also known to bind and activate the orphan G-protein coupled receptor GPR55, which triggers proliferation and anchorage-independent growth of OC cells, as well as activation of Akt and ERK1/2 kinase^17^.

Phosphatidylinositol is one of several inositol membrane phospholipids known to be responsible for recruitment of the serine/threonine kinase Akt to the plasma membrane and its subsequent phosphorylation and activation^18^. Phosphorylation of the inositol ring 3′-OH group in inositol phospholipids is carried out by the enzyme phosphatidylinositol 3-kinase (PI3K). A broad range of functions related to cancer onset and progression have been associated with PI3K activity, including proliferation, cell adhesion, apoptosis, and transformation^19^. Our identification of the sphingolipid ceramide as a differentiating metabolite is consistent with its previously proposed roles in ovarian and other cancers^20^.

## Discussion

As demonstrated by the presented work, SVM machine learning is a powerful computational tool for the identification of correlated patterns in large datasets. We have previously shown that combining this computational approach with high-resolution mass spectrometry of patient sera is a minimally invasive and highly accurate method for the detection of prostate^10^ and late-stage ovarian cancers^21^.

Because of the extensive genetic diversity known to exist among individual patient tumors of even the same type of OC^22^, it is not surprising that it has proven extremely difficult to identify a single set of biomarkers capable of diagnosing the disease with high accuracy^23^. Although there may be multiple genetic lesions and alternative molecular pathways leading to the development of even the same type of OC, all of these mutations and pathways converge on a similar cancer phenotype. Thus, molecular features closely associated with the cancer phenotype, like metabolites, may be expected to be less variable across patients than the broader spectrum of individual mutations and disrupted pathways underlying the disease^24^.

The predictive accuracy of SVM-derived biomarkers is heavily dependent upon the representative nature of the biological samples used in building the model. For this reason, we designed our study to include samples collected from a broad spectrum of geographic locations in the United States and Canada. However, more extensive sampling of patients across a wider diversity of racial and ethnic groups will be needed to determine the general robustness of the diagnostic biomarkers presented here. Regardless, our results demonstrate that this evidence-based approach to metabolic biomarker discovery is conceptually unbiased for the establishment of highly accurate biomarker panels of early-staged OC across a broad geographic area. If deemed appropriate by future studies, the method can be equally well applied to racial or ethnic sub-populations to obtain optimally accurate panels of metabolic features in these cohorts. When combined with experimental chemical identification of these diagnostic features, our approach provides valuable insight into the metabolic alterations accompanying the disease and can serve as the foundation for clinically significant diagnostic tests.

## Methods

### Chemicals

Ultrapure water with 18.2 MΩ cm resistivity (Barnstead Nanopure UV ultrapure water system, USA) was used to prepare all mobile phase components. Chromasolv^®^ (Fluka) LC-MS grade methanol was purchased from Sigma-Aldrich Corp. (St. Louis, MO, USA). Lysophatidylinositol (18:1) and ceramide (d18:1/16:0) were purchased from Avanti Polar Lipids, Inc. (Alabaster, AL, USA).

### Sample Preparation

All samples were collected after informed consent under approved IRB protocols. Serum samples were thawed on ice, and protein precipitation was performed by the addition of methanol in a 3:1 volume ratio to 50 μL of serum. Aliquots of 10 μL from each sample were combined to create a pooled sample, which was split into 50 μL portions before protein precipitation. Samples were vortex-mixed for 10 s and centrifuged at 13,000 g for 7 min. After centrifugation, 150 μL of supernatant was mixed with 400 μL of ultrapure water prior to solvent removal using a VirTis benchtop freeze dryer (Warminster, PA). Samples were stored at −80 °C until analysis. Samples were separated into 8 batches with equal representation of epithelial ovarian cancer (EOC) and control samples from each collection site in each group. All samples were thawed, reconstituted with 80:20 (v:v) H_2_O: MeOH, and analyzed in duplicate. Samples were run in alternating fashion so that duplicate runs for a specific sample were not consecutive. Pooled quality control serum samples were analyzed every eight sample runs. The mass spectrometer was mass calibrated before analysis; and solvent, sample preparation blanks, and pooled samples were analyzed jointly with the EOC and control samples.

### UPLC-MS

UPLC-MS was performed using a Waters ACQUITY Ultra-Performance LC system (Waters Corporation, Manchester, UK), fitted with a Waters ACQUITY UPLC BEH C18 column (2.1 × 50 mm, 1.7 μm particle size), coupled to a high-resolution accurate mass Synapt G2 high-definition mass spectrometry system (Waters Corporation, Manchester, UK). The Synapt G2 HDMS is a hybrid quadrupole-ion mobility-orthogonal acceleration time-of-flight instrument with a typical resolving power of 20,000 FWHM and mass accuracy of 9 ppm at m/*z* 544.2615. The instrument was operated in negative ion mode with a probe capillary of 2.0 kV and a sampling cone voltage of 35 V. The source and desolvation temperatures were 150 and 500 °C, respectively, and the nitrogen desolvation flow rate was 1000 L h^−1^. The mass spectrometer was calibrated across the range of m/*z* 50–1200 using a 0.5 mM sodium formate solution prepared in 90:10 (v/v) 2-propanol:water. Data were mass corrected during acquisition using a leucine enkephalin reference spray (LockSpray) infused at 3 μL min^−1^. Data were acquired in the 50–1200 m/*z* range, and the scan time was set to 1 s. Data acquisition and processing were carried out using MassLynx V4.1 and MZmine V2.0, respectively. The chromatographic method for sample analysis involved elution with ultrapure water (mobile phase A) and methanol (mobile phase B) using the following gradient program: 0–15 min 80–10% A; 15–23 min 10% A. The flow rate was constant at 0.40 mL min^−1^ for 23 min. The gradient was returned to its initial conditions with a solvent blank run of 11 min. The column temperature was set to 60 °C, the autosampler tray was set to 5 °C, and the injection volume was 8 μL. UPLC-MS/MS experiments were performed by acquiring mass spectra with applied voltages between 5 and 50 V in the trap cell, using ultra high purity grade argon (>=99.999%) as the collision gas.

### Data processing/analysis

Metabolic features (retention time (R_t_), m/*z* pairs) were extracted from chromatograms using MZmine V2.0 software and Excel. A five point Savitzky Golay smoothing function was applied to each scan of the raw data prior to peak detection. After chromatogram alignment, the subsequent peak list was conservatively filtered by elimination of peaks that were not present in at least 40 of the 237 collected runs prior to gap filling. The exported peak areas for each sample were normalized by division of the total peak area sum for that sample in Excel. Potential features in which the slope of the peak area of the pooled samples vs. time changed more than one standard deviation away from zero were removed from the peak list. The potential features list was further constrained by purging features that were not present in 50% of sample groups (all samples, EOC samples, or control samples) at ten times the baseline, defined as the maximum peak area observed in the sample blank and ten mobile phase runs (one from each day of analysis). The duplicate sample peak areas were averaged to create a matrix containing sample peak areas for each feature (average R_t_, average m/*z*).

The data set was scrutinized for the presence of experimental and instrument bias with principal component analysis (PCA) using MATLAB R2012b (Version 8.0.0.783 The MathWorks, Inc., Natick, MA, USA) and the PLS Toolbox (v.6.71, Eigenvector Research, Inc., Wenatchee, WA, USA). Peak area data were labeled with the corresponding collection day, analysis batch, or sample origin. Data were preprocessed by autoscaling, and PCA run with leave-one-out cross-validation. Sample clustering was assessed with the plot of the first versus second principal component.

Linear support vector machine (SVM) analysis of the feature matrix was performed with in-house-developed code utilizing liblinearSVM^25^. Recursive feature elimination (RFE) was used to find the minimum set of discriminant features that maximized accuracy in the classification^9^. For a binary classification problem, linearly-separable samples represented as a row vector ***x***, had membership of two classes *g* (=N or C), where N stands for normal or control patient samples and C represents EOC disease patient samples with class value *c* (=−1 for class N, and +1 for class C). The decision function that separated the two classes, defined here as the “EOC metabolic score”, was as follows:


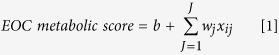


where *w* and *b* are the weight and bias parameters that were determined from the training set and J is the total number of features. The sign of the EOC metabolic score determined which class a sample was assigned to: class N if negative and class C if positive. In this classification function, the two classes were divided in the dataspace by a hyperplane ***wx**’* + *b* = *0* that maximized the margins between samples of different classes. The margin between the two classes was defined such that:


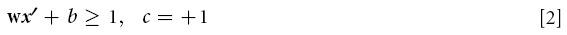



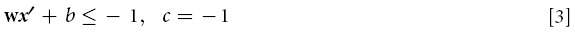


The RFE method involved an “outer” matrix with 95 columns (equal to the number of serum samples) and 255 rows (equal to the number of features). Each row in the outer matrix represents a subset of features to be tested for discriminatory power. Each subsequent row examines a feature subset that contains one less feature than the previous row. For each row, a set of 95 “inner” matrices is constructed, each one containing a subset of samples to build an SVM model and one sample left out for testing, following standard LOOCV practice. After model building, the SVM feature weights were calculated for each inner matrix, and these weights were summed across the inner matrix. The average feature weight was then calculated across the outer matrix row. The least important feature was then discarded. This process was subsequently repeated for every outer matrix row. A panel of optimal features was determined by examining which feature set had maximized accuracy, sensitivity, and specificity. Data were preprocessed by autoscaling the features across the samples prior to SVM-RFE.

Orthogonal partial least squares discriminant analysis (oPLS-DA^9^) was performed to inspect data after discriminant feature selection via SVM-RFE. oPLS-DA models were internally cross validated using leave-one-out, venetian blinds (10 data splits and 10 samples per blind), contiguous block (10 splits), or random subsets (10 data splits and 10 iterations) approaches. Permutation testing was performed by randomizing the class labels for all samples. Data were preprocessed by autoscaling the features’ peak areas across the samples.

### Metabolite Identification

Compound identification was carried out for the 16 discriminant features obtained after the feature selection processes. Elemental formulas were generated based on the mass accuracy of the peak of interest and isotopic patterns with a mass error of 10 mDa using MassLynx 4.1. The chemical formulas were searched against the following publically-available databases: Metlin, the human metabolome database (HMDB), Metabolomics Workshop, LIPID Metabolites and Pathways Strategy (LIPID MAPS), and MassBank to determine possible endogenous metabolite candidates. Entries in the MS/MS Metlin database, MassBank, and Lipid Maps, together with literature searches subsequently confirmed the identity of putative candidates. When available, metabolite standards were analyzed to support identification. Identification of metabolites was pursued according to established criteria^26^.

## Additional Information

**How to cite this article**: Gaul, D. A. *et al.* Highly-accurate metabolomic detection of early-stage ovarian cancer. *Sci. Rep.* **5**, 16351; doi: 10.1038/srep16351 (2015).

## Supplementary Material

Supplementary Information

## Figures and Tables

**Table 1:** Chemical identification of 16 features that distinguish early-staged ovarian cancer sera from the sera of normal healthy controls with high accuracy (m/*z* = mass-to-charge ratio, min = minutes, ppm = part per million, CAS = chemical abstract service, USPTO = United States Patent and Trademark Office, HMDB = the human metabolome database, SVM = support vector machine, NA = not available).

Feature	Average m/*z*	Average Retention Time (min)	Ion Type	Ion Theoretical m/*z*	Mass Error (ppm)	Neutral Elemental Formula	Tentative Metabolite Identification [Database: #]	SVM Model Weight
279	552.2327	0.70	[M-H]^2−^	552.2335	−1.4	C_42_H_80_N_3_O_30_	[NA]	1.1521
				552.2342	−2.7	C_43_H_76_N_7_O_26_		
				552.2398	−12.9	C_42_H_76_N_9_O_25_		
571	329.1733	4.81	[M-CHO-H]^−^	329.1753	−6.1	C_21_H_28_O_5_	cortisone [CAS: 53-06-5]	1.0810
286	597.3029	10.89	[M-H]^−^	597.3040	−1.8	C_27_H_51_O_12_P	lysophatidylinositol(18:1) [CAS: 1246298-13-4]	0.4762
683	261.0727	1.01	[M-H]^−^	261.0723	1.5	C_9_H_13_N_2_O_7_	aspartyl-glutamic acid [CAS: 6157-06-8]	0.3967
226	539.4301	14.86	[M-H]^−^	539.4312	−2.0	C_32_H_60_O_6_	16-(6-butoxy-3-hydroxy-4,5-dimethylcyclohex-1-en-1-yl)-6,10-dihydroxy-2,6,10,14-tetramethyl hexadecanoic acid [USPTO: document #20100086960]^a^	0.1748
45	536.5042	18.56	[M-H]^−^	536.5043	−0.2	C_34_H_67_NO_3_	ceramide(d18:1/16:0) [CAS: 24696-26-2]	0.1076
64	365.3413	16.97	[M-H]^−^	365.3425	1.2	C_24_H_46_O_2_	[NA]	0.0847
28	524.2778	12.79	[M-H]^−^	524.2777	0.2	C_27_H_44_NO_7_P	lysophosphatidylethanolamine(22:6) [PUBCHEM: 52925132]	0.0445
105	195.1016	1.01	[M+Na-2H]^−^	195.0997	9.7	C_9_H_18_O_3_	2-hydroxyl nonanoic acid [CAS: 617-31-2]^a^	0.0195
14	307.2633	14.80	[M+Na-2H]^−^	307.2613	6.5	C_18_H_36_O_2_	iso-1,2-octadecanediol [PUBCHEM: 42607317]^a^	−0.0200
79	245.1378	0.99	[M-H]^−^	245.1389	−4.5	C_12_H_22_O_5_	3-hydroxyl dodecanedioic acid [CAS: 34574-69-1]^a^	−0.0205
80	883.5358	15.40	[M-H]^−^	883.5337	2.4	C_47_H_81_O_13_P	phosphatidylinositol(20:4/18:1) [HMDB: 09901]	−0.0308
123	467.3727	14.61	[M-H]^−^	467.3737	−2.1	C_28_H_52_O_5_	7,9,13-trihydroxyoctacosa-16,22-dienoic acid [USPTO: document #20120136057]^a^	−0.0803
231	429.2997	11.49	[M-H]^−^	429.3010	1.3	C_27_H_42_O_4_	[NA]	−0.3420
261	451.2275	1.58	[M-H]^2−^	451.2242	7.3	C_40_H_68_N_6_O_17_	[NA]	−0.4834
				451.2249	5.8	C_41_H_64_N_10_O_13_		
				451.2305	−6.6	C_40_H_64_N_12_O_12_		
620	129.0909	1.14	[M-H]^−^	129.0916	−2.1	C_7_H_14_O_2_	[NA]	−0.9938

^a^All possible metabolite isomers are not listed. Where indicated, the species in the Table are those for which MS/MS data was available in the literature.
