# Supplementary material for: Highly-accurate metabolomic detection of early-stage ovarian cancer
Source: Sci Rep. 2015 Nov 17;5:16351. doi: 10.1038/srep16351 (PMC4647115; doi:10.1038/srep16351)
Supplement: Supplementary Information [file srep16351-s1.pdf]

Highly-accurate metabolomic detection of early-stage ovarian cancer

David A. Gaul[1,2], Roman Mezencev[2], Tran Q. Long[3], Christina M. Jones[1], Benedict B. Benigno[4], Alexander Gray[3], Facundo M. Fernández[1,5,*] John F. McDonald[2,4,5*]

[1]School of Chemistry and Biochemistry, Georgia Institute of Technology, Atlanta GA 30332 (USA).

[2]School of Biology, Integrated Cancer Research Center, Georgia Institute of Technology, Atlanta GA 30332 (USA).

[3]College of Computing, Georgia Institute of Technology, Atlanta GA 30332 (USA).

[4]Ovarian Cancer Institute, Atlanta GA 30342 (USA).

[5]Parker H. Petit Institute of Bioengineering and Biosciences, Georgia Institute of Technology, Atlanta GA 30332 (USA).

Correspondence should be addressed to J.F.M. (john.mcdonald@biology.gatech.edu)

*Equally contributing authors

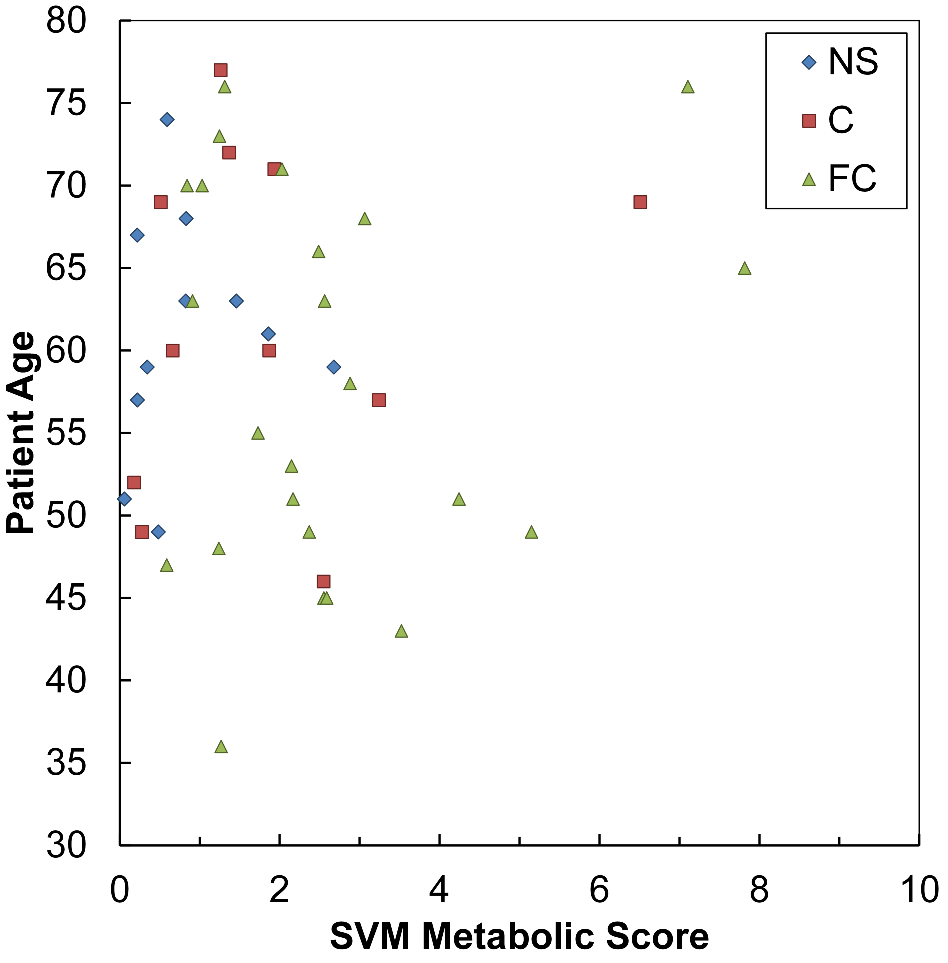

Fig. S1. SVM scores plotted as a function of patient age for each of three patient collection sites. The results demonstrate no clustering of patients based on collection site (NS = Northside Hospital, Atlanta, GA, USA; C = Alberta Health Services, Alberta, Canada; FC = Fox Chase Cancer Center Biosample Repository Facility, Philadelphia, PA, USA).

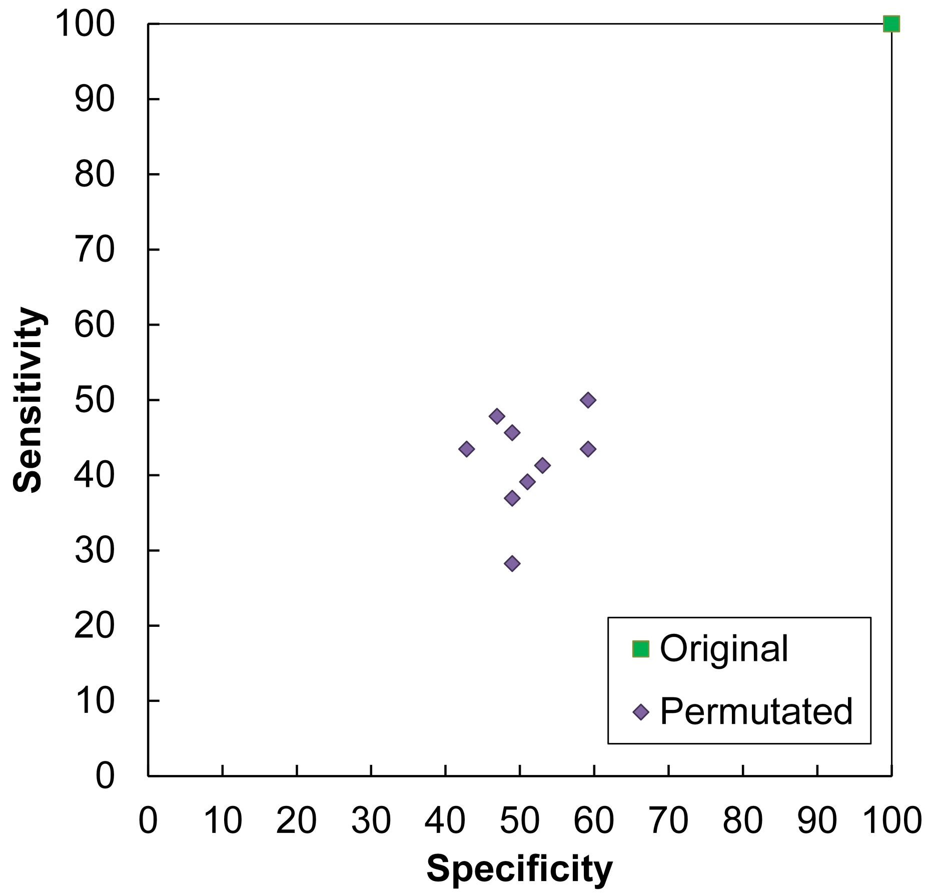

Fig. S2. **Permutation test (n=10) for SVM model built incorporating 16 selected features.** The class labels were randomized prior to building the SVM models. Plot of sensitivity versus specificity illustrates the class label dependence. The significant separation between the original and permutated data points indicates that the original model was not over fit.

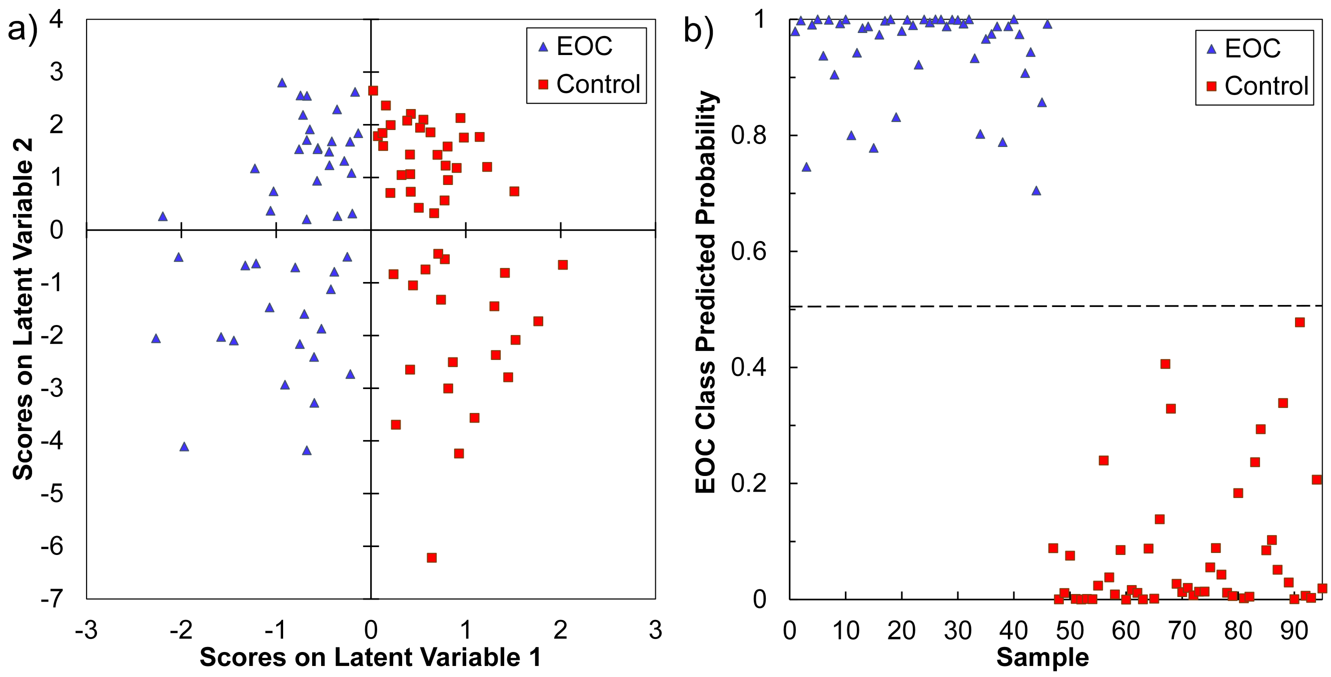

Fig. S3. oPLS-DA model built using the selected 16 features identified from SVM-RFE. oPLS-DA models were internally evaluated by leave-one-out cross validation (LOOCV). Five latent variables were found to capture 54.4% of the X-block variance (feature peak areas) and 34.9% of the Y-block variance from the (class membership). The LOOCV validated accuracy, sensitivity, and specificity were 96.8%, 95.7%, and 98.0%, respectively. a) Score plot of first and second latent variables from the oPLS-DA model built with LOOCV illustrating sample separation into EOC (triangles) and control (square) classes. b) The predicted probability of EOC class membership, where 1 is most probable and 0 is least likely.

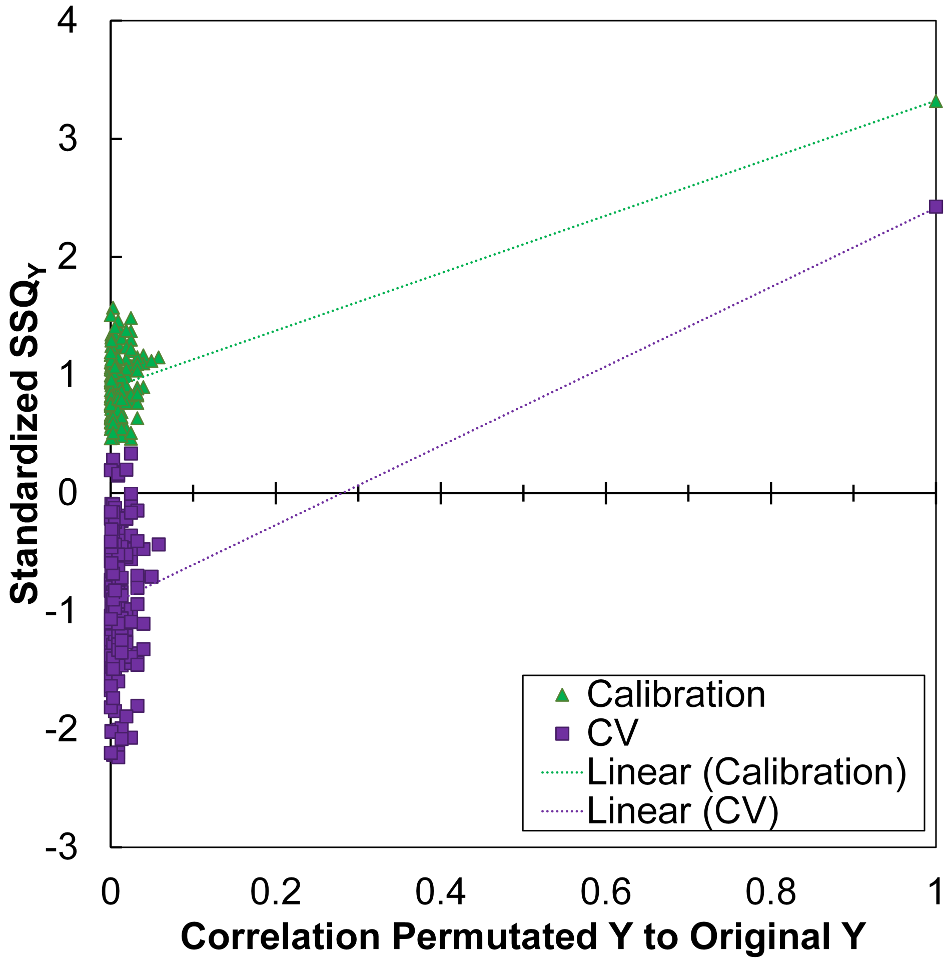

Fig. S4. Y-block permutation results for 200 iterations for both self-predicted (calibration, C) and cross-validated (CV) values. Standardized sum squared Y (SSQ_Y) was plotted versus correlation of permutated results to un-permutated results. The original un-permutated results are shown on the right side of the figure. The significant separation between permutated (left side) and un-permutated (right side) results along with higher y values for the permutated versus the un-permutated results support that the original model is more likely significant, and <u>not</u> over fit.

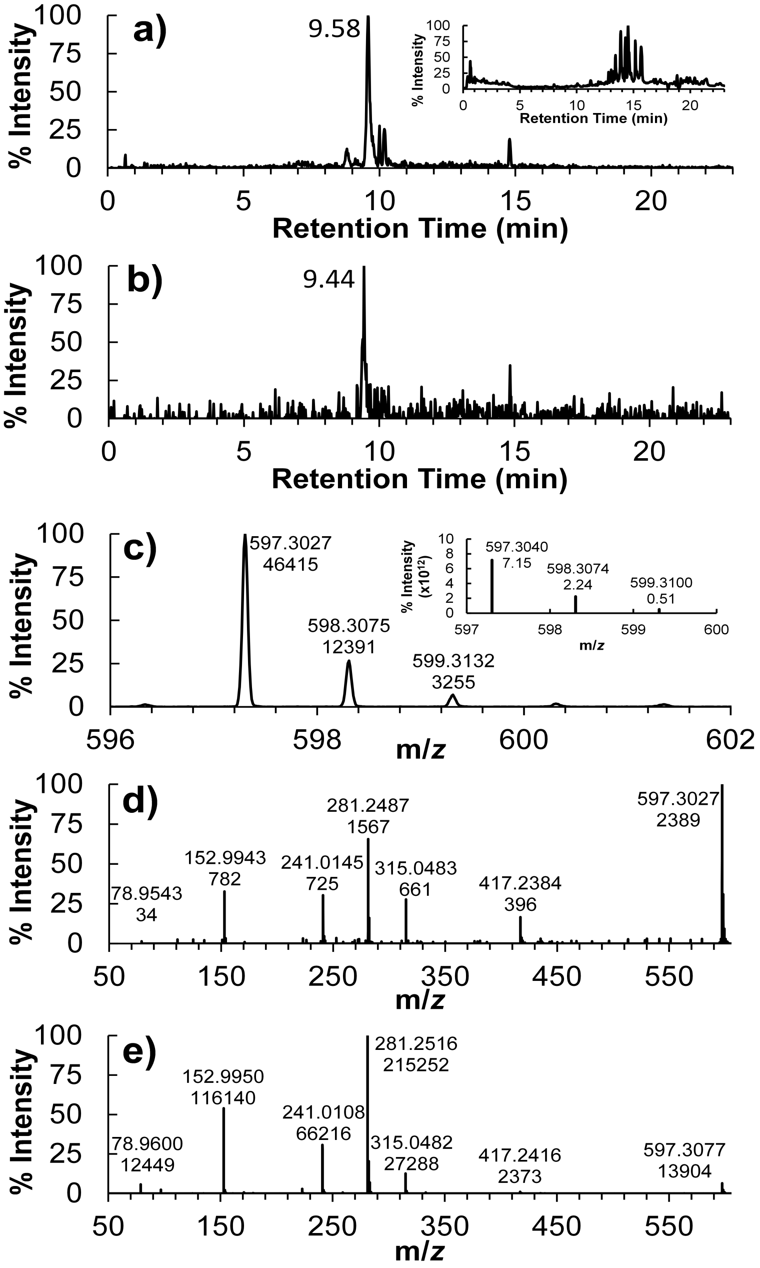

Fig. S5. Identification of metabolite #286 as lysophosposphatidylinositol (18:1). (a) Extracted ion chromatogram of m/z 597.3029 from a pooled serum sample (inset spectrum is total ion chromatogram from a pooled serum sample); (b) extracted ion chromatogram of m/*z* 597.3029 from a standard; (c) experimental isotopic pattern that agrees with theoretical isotopic pattern for $C_{27}H_{51}O_{12}P$ (see inset spectrum, calculated using MassLynx V4.1); d) MS/MS spectrum from a pooled serum sample collected using a collision energy (CE) of 30 eV; and e) MS/MS spectrum (CE 30 eV) of direct injection of standard.

Table S1. **Histopathologies of patients analyzed in this study.** Listed are FIGO stage and grade (when available) for the cancer patients (normal samples also listed). NS = Northside Hospital Atlanta, GA, USA; C = Alberta Health Services, Alberta, Canada; FC = Fox Chase Cancer Center Biosample Repository Facility, Philadelphia, PA, USA. Average age of EOC patients at blood draw was 59.7 years old (median = 60 years) and the average age of control patients was 56.9 years old (median = 55). The difference in average ages was not significant by T test.

Patient ID	Ovarian Histopathology	FIGO Stage / grade	Age at Blood Draw	Source
1117	Papillary serous carcinoma	2B / 3	68	NS
1105	Papillary serous carcinoma	1C / 1	67	NS
989	Papillary serous carcinoma	1B / 3	51	NS
876	Papillary serous carcinoma	2A / 1	63	NS
550	Papillary serous carcinoma	2A / 2	49	NS
517	Papillary serous carcinoma	1A / 3	59	NS
491	Papillary serous carcinoma	1 / unk	74	NS
336	Papillary serous carcinoma	1C / 3	63	NS
317	Papillary serous carcinoma	1C / 3	59	NS
170	Papillary serous carcinoma	2A / 2	57	NS
2	Papillary serous carcinoma	2B / 3	61	NS
RT42	Papillary serous carcinoma	1C / unk	69	C
F5223	Papillary serous carcinoma	2C / 3	52	C
RT68	Papillary serous carcinoma	2C / 3	46	C
RT332	Papillary serous carcinoma	1A / 1	49	C
NEO1	Papillary serous carcinoma	1C / unk	71	C
RT514	Papillary serous carcinoma	1C / 3	57	C
RT965	Papillary serous carcinoma	2A / unk	60	C
RT523	Papillary serous carcinoma	1C / 3	77	C
RT261	Papillary serous carcinoma	2A / 3	72	C
RT36	Papillary serous carcinoma	1B / 3	69	C
RT01	Papillary serous carcinoma	2C / 3	60	C
106220	Papillary serous carcinoma	1C / 3	58	FC
108403	Papillary serous carcinoma	2C / unk	76	FC
111079	Papillary serous carcinoma	1A / 1	47	FC
113885	Papillary serous carcinoma	2C / 2	53	FC
115403	Papillary serous carcinoma	2C / 3	55	FC
116804	Papillary serous carcinoma	1C / 2	68	FC
118020	Papillary serous carcinoma	2A / 3	51	FC
119189	Papillary serous carcinoma	2C / 3	51	FC
119652	Papillary serous carcinoma	1A / 2	43	FC
120136	Papillary serous carcinoma	1A / 1	49	FC
122955	Papillary serous carcinoma	2C / 3	45	FC
123534	Papillary serous carcinoma	2C / 3	66	FC
124093	Papillary serous carcinoma	2B / 3	63	FC

Patient ID	Ovarian Histopathology	FIGO Stage / grade	Age at Blood Draw	Source
125989	Papillary serous carcinoma	1C / 3	49	FC
126530	Papillary serous carcinoma	2B / 3	70	FC
127366	Papillary serous carcinoma	2C / 3	70	FC
127305	Papillary serous carcinoma	2B / 3	71	FC
131043	Papillary serous carcinoma	1A / 3	76	FC
128426	Papillary serous carcinoma	2B / unk	36	FC
129614	Papillary serous carcinoma	1C / unk	45	FC
130196	Papillary serous carcinoma	2B / 3	65	FC
131589	Papillary serous carcinoma	2C / 3	48	FC
133011	Papillary serous carcinoma	2C / 3	63	FC
135520	Papillary serous carcinoma	2C / 3	73	FC

Patient ID	Age at Blood Draw	Source
1094	50	NS
1080	49	NS
1064	63	NS
1051	45	NS
917	49	NS
908	78	NS
875	47	NS
783	52	NS
757	84	NS
755	75	NS
751	60	NS
691	70	NS
682	53	NS
677	68	NS
665	84	NS
645	61	NS
636	71	NS
627	59	NS
615	42	NS
593	58	NS
592	48	NS
557	61	NS
544	49	NS
540	59	NS
534	72	NS
504	48	NS
494	51	NS

Patient ID	Age at Blood Draw	Source
481	63	NS
479	55	NS
448	63	NS
440	50	NS
1043	52	NS
1046	49	NS
1022	58	NS
960	43	NS
899	45	NS
891	52	NS
873	62	NS
870	40	NS
849	64	NS
842	70	NS
838	79	NS
482	44	NS
719	55	NS
648	50	NS
572	40	NS
524	45	NS
522	61	NS
505	42	NS

Table S2. List of 255 metabolic features (R_t, m/*z* pairs) used to build a discriminant linear support vector machine (SVM) model to distinguish sera from epithelial ovarian cancer patients vs. normal controls.

Feature	m/z, R_t
342	125.0960, 1.81
284	125.0963, 1.15
620	129.0909, 1.14
579	157.1222, 2.29
140	171.1380, 3.49
280	177.1267, 1.96
12	187.0068, 0.69
74	187.0962, 0.72
378	187.0965, 0.99
438	191.0685, 1.78
105	195.1016, 1.01
186	203.0821, 0.60
490	209.0799, 1.69
170	221.1265, 2.24
403	223.1329, 1.23
539	224.0589, 1.03
41	224.0591, 0.84
379	225.0630, 0.86
43	225.1844, 11.68
1229	226.1802, 6.92
615	227.1161, 2.69
631	229.1243, 1.62
633	231.1115, 2.20
59	238.0751, 0.93
125	239.0879, 0.99
271	241.0859, 1.59
79	245.1378, 0.99
243	251.2001, 12.66
257	253.1349, 3.44
6	253.2165, 13.35
260	254.6201, 2.56
174	254.6201, 2.55
92	255.6277, 3.60
410	259.1540, 1.30

Feature	m/z, R_t
683	261.0727, 1.02
873	265.1466, 8.17
88	265.1469, 7.79
406	267.1231, 1.23
129	271.2271, 12.06
50	273.1678, 1.78
229	275.2002, 12.52
419	279.6111, 2.65
274	280.6188, 3.48
25	281.3435, 14.51
673	282.1439, 1.34
641	283.1511, 1.48
230	285.1231, 3.44
604	287.1854, 2.49
130	289.1625, 1.14
238	291.122, 3.43
264	291.1229, 1.62
987	294.6169, 6.45
1016	295.2259, 10.96
1648	297.1513, 8.52
319	301.2005, 3.53
84	301.2164, 13.13
639	302.1125, 1.62
285	302.1126, 3.46
8	303.2324, 13.76
35	305.2476, 14.28
14	307.2633, 14.80
16	309.2789, 15.31
1238	310.1561, 3.82
58	311.1392, 1.65
277	311.6628, 2.78
223	311.6629, 2.76
269	313.1038, 3.45
156	313.1181, 5.29
630	313.1184, 4.94
13	327.232, 13.71
581	328.1576, 7.52
571	329.1733, 4.81
15	329.2477, 14.13
107	331.1898, 4.89

Feature	m/z, R_t
10	339.2303, 13.54
83	353.1615, 2.12
64	365.3413, 16.97
55	367.1573, 2.95
546	369.1727, 3.31
70	369.1731, 3.98
547	369.1732, 4.57
429	383.1525, 1.84
611	385.1671, 1.32
418	388.2509, 10.70
121	397.2038, 4.10
608	399.2179, 6.36
132	407.2192, 12.29
149	409.2347, 13.18
114	409.2348, 12.96
952	411.4854, 0.65
564	413.1989, 3.23
71	415.3568, 16.88
231	429.2997, 11.49
27	429.373, 17.38
131	433.2348, 12.56
100	433.2349, 12.75
202	435.2508, 13.52
1086	445.3309, 14.72
96	445.3313, 14.75
599	447.1329, 6.11
159	447.3469, 14.92
127	448.3058, 8.04
181	449.3618, 15.14
261	451.2275, 1.58
541	452.2772, 11.89
19	452.2776, 13.23
478	465.3544, 13.11
123	467.3727, 14.61
241	473.3621, 15.10
124	476.2769, 12.71
24	476.2777, 12.81
180	478.2931, 12.29
31	478.2934, 13.50
161	480.3084, 13.18

Feature	m/z, R_t
138	480.3086, 13.05
32	480.3088, 14.23
128	487.2037, 0.69
401	498.2884, 7.37
535	498.9287, 7.46
569	498.9291, 6.99
29	500.2776, 12.81
62	501.3056, 10.35
295	501.3935, 15.57
351	503.4092, 15.94
204	504.3075, 12.64
148	504.308, 12.75
434	506.3255, 13.50
146	508.4724, 17.21
380	508.7409, 0.92
356	515.321, 10.84
340	522.4885, 17.88
28	524.2778, 12.79
110	526.2931, 13.16
66	529.3378, 11.18
196	529.4221, 13.37
310	531.1789, 13.00
144	534.4874, 17.52
45	536.5042, 18.56
473	537.2473, 3.45
957	537.414, 14.59
102	537.4151, 14.63
113	539.43, 14.73
226	539.4301, 14.86
888	540.4332, 14.61
279	552.2327, 0.70
282	555.4542, 16.20
155	557.4571, 17.34
56	557.4572, 16.79
875	558.4605, 16.80
360	561.487, 18.22
188	564.5352, 20.55
189	573.4520, 14.90
207	575.4655, 16.44
198	575.4663, 15.99

Feature	m/z, R_t
201	577.4813, 16.47
108	581.4537, 13.82
137	582.5082, 18.53
76	585.4853, 14.50
337	588.4387, 16.84
309	591.3911, 14.54
171	591.4614, 14.89
48	593.4776, 15.28
154	595.2876, 10.10
34	595.4929, 15.49
256	595.7483, 0.71
286	597.3029, 10.89
267	599.3189, 11.64
531	601.7721, 0.74
329	605.4535, 13.78
175	614.4536, 17.10
206	616.4699, 18.09
33	617.732, 0.65
82	619.2874, 10.13
90	620.5979, 18.20
668	627.3723, 7.62
157	634.6114, 21.26
364	642.4857, 18.36
1011	645.4488, 18.46
381	646.6128, 19.47
213	646.6128, 19.27
160	657.4957, 17.10
795	671.4638, 19.04
798	673.4798, 20.42
195	673.4802, 20.36
884	673.7984, 0.77
1030	685.5277, 18.36
450	688.4908, 18.53
512	695.4644, 18.83
37	696.7672, 0.67
93	698.5111, 19.98
789	698.5113, 20.14
63	698.5568, 17.85
355	699.4957, 21.18
461	700.5245, 21.60

Feature	m/z, R_t
1270	714.5073, 19.07
78	716.522, 20.439
1542	720.4897, 18.71
22	722.5133, 19.74
251	723.4963, 20.93
217	724.5256, 20.72
91	724.527, 20.44
338	726.5389, 22.56
655	726.5422, 22.65
413	729.4412, 12.38
304	731.312, 0.69
583	736.5267, 21.06
344	736.5268, 20.86
232	736.5269, 20.94
388	738.507, 18.37
81	738.5073, 19.05
786	740.5231, 19.53
72	740.5232, 19.38
312	742.5382, 21.16
42	742.5386, 21.24
779	742.5397, 21.59
185	744.562, 17.85
135	746.5117, 19.07
776	746.5122, 19.57
21	746.5127, 19.54
350	747.5636, 18.10
18	748.5287, 20.25
199	750.5408, 20.57
1138	750.5433, 21.99
46	750.5438, 22.41
291	757.4730, 12.99
492	760.5257, 20.46
709	760.5299, 20.40
773	762.5069, 18.77
311	762.5071, 18.28
780	764.522, 19.37
307	764.5221, 19.83
39	764.5237, 19.28
20	766.5394, 21.07
339	768.5528, 22.29

**Feature**	**m/z, R_t**
69	772.5287, 20.02
785	772.5293, 20.07
415	772.5457, 13.17
858	774.5432, 20.88
73	774.5442, 22.12
192	774.5639, 13.45
796	788.5223, 19.12
783	790.5372, 21.11
57	790.5396, 20.88
470	804.5555, 18.66
54	833.5196, 15.26
67	835.5352, 15.56
47	857.5197, 15.25
122	859.5345, 15.41
193	860.6111, 17.58
2009	861.5512, 15.71
211	863.5657, 16.15
80	883.5358, 15.40
77	887.5679, 15.96
182	909.5515, 15.67

Table S3. **oPLS-DA models built with the SVM-RFE selected 16 features incorporating different cross validation (CV) techniques.** Due to limited sample size, data over-fitting is a valid concern. The multivariate SVM-RFA model was thus validated by comparing with oPLS-DA using several cross-validation (CV) approaches, as detailed in the table. Some CV approaches are more stringent than others, but all show consistently high accuracy (>90%). As a negative control, we used a permutation approach showing that when the labels for the samples are scrambled, there is NO classification (*i.e*. the accuracy drops to 50-60%, which is close to a random object draw). Overall, our results demonstrate that regardless of CV strategy employed we did not find any evidence of over-fitting of the selected metabolite abundances. Techniques include: leave-one-out, venetian blinds (10 data splits and 10 samples per blind), contiguous block (10 splits), and random subsets (10 data splits and 10 iterations). The root mean square error (RMSE) is a measure of performance of how close the data are to the model prediction. RMSE scale is 0 to ∞, with 0 being the best correlation. Similarity of RMSE for the calibration and CV suggests the model is not over fitting the data. For comparison, scores from the permutated set (class labels scrambled) are also included below to demonstrate the oPLS-DA model's dependence on the class labels.

	**Accuracy**	**Sensitivity**	**Specificity**	**RMSE EOC**	**RMSE Control**
oPLS-DA calibration	1.000	1.000	1.000	0.557	0.584
Leave-one-out CV	0.968	0.957	0.980	0.599	0.628
Venetian blinds CV	0.915	0.891	0.939	0.622	0.627
Contiguous block CV	0.915	0.891	0.939	0.599	0.625
Random subsets CV	0.947	0.933	0.961	0.603	0.629
oPLS-DA calibration with label permutation	0.658	0.816	0.500	0.696	0.673
Leave-one-out CV with label permutation	0.521	0.694	0.348	0.781	0.751

Table S4. **Detailed Tandem MS characteristics of the panel for 16 metabolites that distinguish cancer and control samples with optimal accuracy.** The fragment ions and the corresponding collision energy (CE) are listed in the table. The selected precursor ions are underlined. Asterisks indicate doubly charged species. The fragments were matched to standards, literature spectra, or were consistent with potential molecular structure. The ions in bold are those matched to standard spectra or literature spectra. Each metabolite was identified according to the following four ID levels: 1) elemental formula, retention time, and MS/MS spectrum of standard matched to feature; 2) MS/MS spectrum consistent with literature spectra and fragmentation ions observed consistent with proposed structure; 3) putative compound class based on chromatographic elution window; and 4) unknown compounds.

Feature ID	CE (eV)	m/z of Fragment Ions Observed in MS/MS Experiments	ID Level	Match Details (Source)
279	20	827.3548, 809.3323, 747.3443, 552.2395*, 543.2249*, 534.2239*, 413.1849*, 273.0027, 259.0977, 241.0892, 184.0645, 167.0386, 127.0412, 113.0359	4	---
571	20	**329.1796**, **311.1724**, **301.1815**, 176.9747, **149.0667**, **137.0574**, 122.0354, **109.0600**	2	consistent with spectrum (Metlin)
286	30	**597.3027**, **417.2384**, **315.0561**, **281.2487**, **241.0145**, **152.9943**, **78.9543**	1	consistent with spectrum from standard, ΔR_t=0.14 min
683	20	261.0698, 217.0877, 182.0152, 173.9687, 162.0230, 145.0616, 122.0789	2	fragmentation consistent with structure
226	30	**539.4307**, **521.4172**, 503.3958, **495.4426**, **477.4211**, **315.2501**, **313.2350**, **297.2489**, **279.2286**, **259.1963**, **223.1738**	2	consistent with spectrum (patent application US20100086960)
45	30	**536.5101**, **506.5003**, **504.4754**, **488.4896**, 308.3023, **296.2623**, **280.2670**, **263.2381**, **254.2506**, **237.2218**	1	consistent with spectrum from standard, ΔR_t=0.12 min
64	40	365.3416, 311.1647, 283.2539, 253.0947, 239.0772, 225.0588, 211.0508, 183.0102, 96.9600	4	---
28	30	**524.2764**, **327.2311**, **283.2466**, 249.1870, 229.1901, **214.0530**, **196.0394**, 177.1667,	2	consistent with predicted

Feature ID	CE (eV)	m/z of Fragment Ions Observed in MS/MS Experiments	ID Level	Match Details (Source)
		**140.0111**, **78.9582**		spectrum (Lipid Maps)
105	20	195.1039, 151.1074, 135.0837, 123.0761	2	fragmentation consistent with structure
14	30	307.2668, 289.2530, 255.2345, 183.0102	2	fragmentation consistent with structure
79	20	245.1385, 187.0961, 169.0847, 125.0972, 123.0809, 97.0633	2	similar to azelaic acid spectrum (MassBank)
80	40	**883.5355**, **601.2842**, 599.3239, 581.3085, **439.2220**, 419.2592, **303.2337**, 283.2613, **241.0078**, **223.0040**, **152.9943**, **78.9582**	2	consistent with predicted spectrum (Lipid Maps)
123	30	**467.3770**, **449.3589**, **423.3873**, **405.3660**, **297.2414**, **279.2286**, **263.2381**, **251.1994**, **223.1607**, **215.1610**, **187.1320**, **169.1188**, **141.1308**	2	consistent with spectrum (patent US20120136057)
231	40	429.3003, 411.2852, 305.2087, 277.2086, 123.0809	4	---
261	20	729.3936, 685.3970, 590.2924, 572.2783, 554.2859,512.2432, 451.2338*, 430.2339*, 400.8628, 374.8882, 330.1878, 312.1372*, 286.1901, 276.6193*, 255.6189*, 173.0992, 131.0891	4	---
620	20	129.0945, 115.9221, 100.9348, 99.9227	4	---
